# Infrared spectroscopy across scales in length and time at BESSY II

**DOI:** 10.1107/S1600577524002753

**Published:** 2024-04-23

**Authors:** Alexander Veber, Ljiljana Puskar, Janina Kneipp, Ulrich Schade

**Affiliations:** a Department of Chemistry, Humboldt-Universität zu Berlin, Brook-Taylor-Straße 2, 12489 Berlin, Germany; b Institute for Electronic Structure Dynamics, Helmholtz-Zentrum Berlin für Materialien und Energie GmbH, Albert-Einstein-Straße 15, 12489 Berlin, Germany; ESRF – The European Synchrotron, France

**Keywords:** infrared beamline, BESSY II, nano-spectroscopy, cellulose microfibrils

## Abstract

The infrared beamline at BESSY II storage ring was upgraded recently to extend the capabilities of infrared microscopy. The endstations available at the beamline are now facilitating improved characterization of molecules and materials at different length scales and time resolutions. Here, the current outline of the beamline is reported and an overview of the endstations available is given. In particular, the first results obtained by using a new microscope for nano-spectroscopy that was implemented are presented. The capabilities of the scattering-type near-field optical microscope (s-SNOM) are demonstrated by investigating cellulose microfibrils, representing nanoscopic objects of a hierarchical structure. It is shown that the s-SNOM coupled to the beamline allows imaging to be performed with a spatial resolution of less than 30 nm and infrared spectra to be collected from an effective volume of less than 30 nm × 30 nm × 12 nm. Potential steps for further optimization of the beamline performance are discussed.

## Abstract 2

The recent upgrade of the infrared beamline at the BESSY II storage ring made possible improved characterization of molecules and materials at different length and time scales. The new nano-spectroscopy endstation based on a scattering-type scanning optical microscope enables infrared imaging and spectroscopy with a spatial resolution better than 30 nm.

## 1. Introduction

The infrared (IR) beamline IRIS at the BESSY II storage ring was inaugurated in 2001 and it is currently the only IR beamline available in Germany for national and international user groups (Peatman & Schade, 2001 ▸; Schade *et al.*, 2002 ▸). The design of the beamline allows broadband high-brilliance radiation spanning from 2 to 10000 cm^−1^ to be extracted. In combination with the top-up operation mode of the BESSY II storage ring (Kuske *et al.*, 2008 ▸), this turns the beamline into a stable radiation source that is well suited for a diverse range of different IR-spectroscopy applications.

The back-end and the endstations are separated from the UHV front-end of the beamline, providing great flexibility in the redistribution of the synchrotron radiation and modification of the endstations. During more than 20 years of operation, the beamline has been constantly developing and evolving to meet the demands of the scientific community, hence offering unique, cutting-edge IR-spectroscopy experimental techniques to the users. Initially equipped with a Fourier-transform infrared (FTIR) spectroscopy and a micro-spectroscopy endstations, the beamline was complemented by an ellipsometer (Gensch *et al.*, 2003 ▸; Hinrichs *et al.*, 2003 ▸) and THz-imaging/spectroscopy set-ups (Schade *et al.*, 2004 ▸) in 2002–2004, instrumentation for microscopy observation of vibrational linear dichroism using polarization-modulated IR synchrotron radiation in 2006–2008 (Schmidt *et al.*, 2006 ▸, 2008 ▸), followed by a dispersive single-shot time-resolved spectrometer (Ritter *et al.*, 2019 ▸) in 2018–2020. During a recent upgrade in 2020–2023, the beamline was extended to fit an additional IR scattering-type scanning near-field optical microscope (s-SNOM). Moreover, imaging capabilities for micro-spectroscopy were extended. In this work, we present the current outline of the beamline and describe the available endstations and their parameters. We discuss and demonstrate the capabilities of the new nano-spectroscopy endstation in more detail. Finally, we discuss the ongoing equipment developments and further modernization of the beamline.

## 2. Beamline design

**Figure 1:**
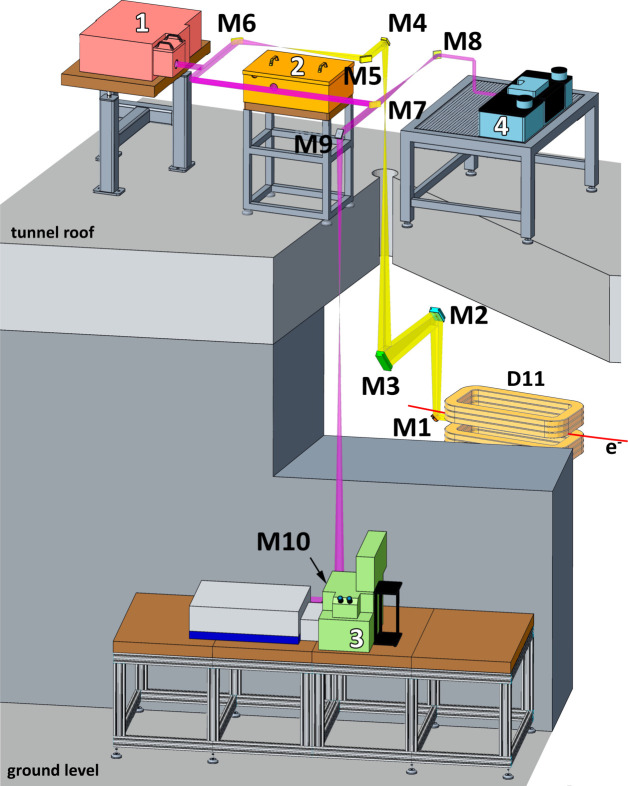
Outline of the current IRIS beamline showing the state as of September 2023. The endstations are marked as follows: (1) IR spectroscopy, (2) single-shot time-resolved IR spectroscopy, (3) IR micro-spectroscopy and (4) IR nano-spectroscopy.

Fig. 1 ▸ displays the current outline of the beamline. The front-end of the beamline has been described elsewhere (Peatman & Schade, 2001 ▸; Schade *et al.*, 2002 ▸). In brief, a slotted mirror (M1) is used to extract the optical radiation originating from the homogeneous region of the bending magnet. After the optical beam is refocused twice with two sets of cylindrical mirrors (M2–M3 and M4–M5). Thereafter the beam is collimated by a toroid mirror (M6) and can be redirected to the different endstations. The beam is not divided into parts and the entire optical radiation is delivered to the respective selected endstation.

The optical scheme extracts the IR beam upwards to the top of the storage ring. Previously, all the endstations were mounted on the monolithic concrete roof of the storage ring, which guaranteed high mechanical stability of the equipment. Due to space constraints, the additional nanospectroscopy endstation would not have fitted on the tunnel roof, therefore the beamline was extended to the ground level of the storage ring experimental hall and an FTIR microscope was accommodated there (Fig. 1 ▸, port 3). The vacuum and optical systems were modified to provide four optical ports: three ports on the roof (Fig. 1 ▸, ports 1, 2 and 4) and one additional at ground level (Fig. 1 ▸, port 3). The location of ports 1 and 2 remained unchanged, and they are currently used for spectroscopy and single-shot time-resolved spectroscopy end­stations, respectively. The micro-spectroscopy endstation, previously also located on the roof, was relocated to ground level (port 3), whereas the space at the roof was used for the nano-spectroscopy endstation (port 4). To re-collimate and deliver the IR beam to port 3 at ground level and port 4 on the tunnel roof, two additional pairs of toroidal mirrors and a couple of plane mirrors were added to the original optical scheme of the beamline. Each mirror reflects the light by 45°. The mirror pairs M7–M8 and M9–M10 re-collimate and resize the beam with a magnification factor of 0.6 and 1.6 for ports 4 and 3, respectively. The magnification factors were chosen to fit the optical input of the corresponding nano- and micro-spectroscopy endstations.

## 3. Endstations of the beamline

### 3.1. IR macro-spectroscopy

Currently, four endstations are available to the users of the beamline. Information about the endstations, the corresponding spectral ranges, available accessories and the measurement modes are listed in Table 1 ▸. The IR macro-spectroscopy endstation is based on a Bruker Vertex 70v vacuum FTIR spectrometer (Bruker, Leipzig, Germany) and is meant mostly for the investigation of macroscopic samples. The measurements with this spectrometer can be performed in the spectral range 20–10000 cm^−1^ in the incoherent multi-bunch synchrotron operational mode. A special low-α operational mode of the synchrotron results in coherent synchrotron radiation at sub-terahertz frequencies, which allows the spectral range at the IR beamline to be extended and high-quality data in the spectral range down to 3 cm^−1^ to be collected (Abo-Bakr *et al.*, 2003 ▸; Schade *et al.*, 2007 ▸; Puskar & Schade, 2016 ▸). This endstation provides the widest spectral range as well as the most variation of the sample environment, including a wide temperature range from 4 to 800 K, controlled gas and humidity atmosphere, and multiple measurement modalities suitable for the investigation of solid and liquid materials. In addition to steady-state measurements, the spectrometer also allows one to perform time-resolved experiments using the step scan and the rapid scan techniques. The endstation is well suited for the precise characterization of novel materials, the study of phase transitions, chemical reactions, *etc*.

### 3.2. Single-shot time-resolved IR spectroscopy

A dedicated spectrometer was implemented for the investigation of non-reversible processes or processes with slow recovery kinetics (Schade *et al.*, 2014 ▸; Ritter *et al.*, 2019 ▸), specifically in studies of protein structure and dynamics. The experimental setup exploits a home-built dispersive IR spectrometer based on a Féry prism, which allows the collection of single-shot spectra in the spectral range 1000–1800 cm^−1^ with a spectral resolution of 1 to 5 cm^−1^ and a time resolution down to about 5 µs. The spectral resolution of the Féry spectrometer is determined by the size of the entrance aperture. The high-brilliance synchrotron radiation enables a 20 µm aperture to be used, which results in the high spectral resolution of the spectrometer. The minimal time resolution is limited by the focal plane array detector used. For a detailed description of the Féry interferometer, refer to our previous publications (Schade *et al.*, 2014 ▸; Ritter *et al.*, 2019 ▸). The current implementation of the endstation allows for studies of photo-induced conformational changes. The performance of the instrument was demonstrated previously on both the irreversible activation of vertebrate rhodopsin and slow-cycling microbial actinorhodopsin systems initiated by a laser pulse of 532 nm (Ritter *et al.*, 2019 ▸).

### 3.3. Upgrade of the micro-spectroscopy endstation

A Continuum microscope coupled to a Nexus 670 spectrometer (Nicolet, Madison, WI, USA), which had been in use for over two decades, was replaced by a Hyperion 3000 IR microscope and a Vertex 80 FTIR spectrometer (Bruker Optics GmbH, Ettlingen, Germany). The new microscope ensures stable in time operation, and the experiments in the mid-IR spectral range can be carried out with the use of a single-point mercury cadmium telluride (MCT) detector or an MCT 64 × 64 pixel focal plane array (FPA) detector, which allows measurements to be performed faster over larger areas of interest. The vibrational linear dichroism modality (Schmidt *et al.*, 2006 ▸) was successfully transferred to the new microscope. The step scan and the rapid scan techniques make the microscope suitable for time-resolved experiments.

**Figure 2:**
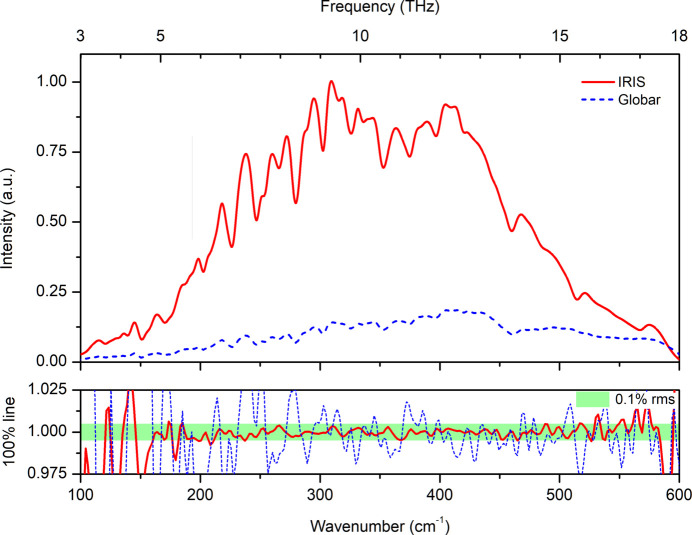
Comparison of the fluxes in the far IR spectral range at the micro-spectroscopy endstation. Using the IR synchrotron radiation the signal intensity is about one order of magnitude higher than using the internal Globar source. An r.m.s. noise of better than 0.1% is achieved from the synchrotron source in the range 150–550 cm^−1^, as indicated by the 100% line, the ratio of two subsequently recorded spectra, each spectrum with an average of 128 individual interferometric scans. The signal is recorded with the square knife aperture set to 100 µm × 100 µm using the Si bolometer.

The new microscope has an optical port for an Si-bolometer, which expands the spectral range available for the endstation in the low wavenumber range to about 80 cm^−1^. The advantage of the synchrotron in experiments with diffraction-limited spatial resolution in the far-IR/THz spectral range is evident from Fig. 2 ▸, comparing the signal intensity of the synchrotron and a Globar source measured through a 100 µm × 100 µm aperture. The use of the synchrotron radiation results in about a one order of magnitude benefit in comparison with the internal Globar source. An r.m.s. noise of better than 0.1% is achieved in the range from 150 to 550 cm^−1^, as indicated by the 100% line, calculated as the ratio of two subsequently recorded spectra (Fig. 2 ▸, bottom panel).

### 3.4. Nano-spectroscopy endstation

The nano-spectroscopy endstation is based on a neaScope scattering-type near-field optical microscope (attocube, Haar, Germany). The beamline radiation is coupled to the near-field spectroscopy module of the microscope, which is based on an asymmetric Michelson interferometer, with an atomic force microscope (AFM) placed in one arm of the interferometer (Knoll & Keilmann, 1999 ▸; Hillenbrand *et al.*, 2002 ▸; Keilmann & Hillenbrand, 2004 ▸; Amarie *et al.*, 2009 ▸; Bechtel *et al.*, 2020 ▸). The incoming collimated radiation is focused with a parabolic mirror (numerical aperture 0.46, *f* = 11 mm) on the tip of the AFM probe, and the interferogram is detected by a liquid-nitro­gen-cooled MCT detector that has an active area of 50 µm × 50 µm and a cut-off at ∼625 cm^−1^ (Infrared Associates, FL, USA). The path of the beam from the vacuum system to the microscope and the microscope itself are purged with dry N_2_ and the microscope is protected by an acoustic enclosure. The current configuration of the microscope allows one to perform measurements in the spectral range 600–2000 cm^−1^, determined by the detector, beam splitter, AFM probe and the incoming synchrotron radiation. The IR synchrotron light can be used for both imaging and point spectroscopy experiments. Using the broadband synchrotron light, imaging is performed at the white-light position of the interferometer and the observed contrast in the recorded images does not contain exact spectral information related to a specific band but rather represents intensity changes over the whole broad spectrum. The point spectroscopy method should be used to reveal the spectral changes at the points of interest. In addition to the synchrotron-based modes, the endstation allows pseudo-heterodyne IR imaging to be performed using an additional tunable single-frequency laser source.

**Figure 3:**
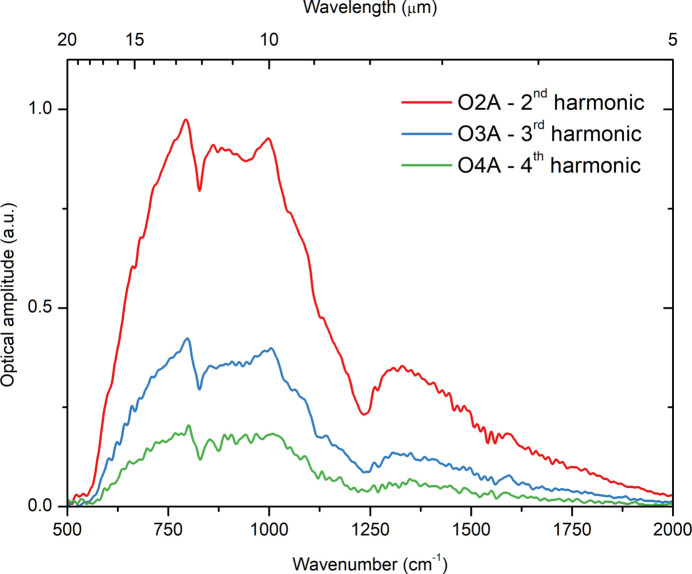
Different harmonics of the optical amplitude signal collected from the Si-reference sample at the nano-spectroscopy endstation: the red/top trace, blue/middle trace and green/bottom trace correspond to the signal’s second, third and fourth harmonic, respectively. The spectral resolution of the spectra is about 8 cm^−1^ and the acquisition time was about 15 min. The AFM probe oscillation frequency is 250 kHz and the tapping amplitude is 75 nm. The high resonant frequency of the AFM probe and the high-frequency cut-off of the ADC at about 1 MHz do not allow the fifth harmonic of the signal to be registered.

The optical amplitude spectrum measured from an Si reference sample and the broadband synchrotron radiation is shown in Fig. 3 ▸. It is known that the contribution from the near-field signal to the overall signal detected by the scattering-type near-field microscope increases with the number of harmonic of the modulation frequency. At least registration of the second harmonic of the optical signal is necessary for a sufficient suppression of the background contribution and extraction of the near-field component from the input signal. The intensity of the optical signal is usually the factor limiting the registration of the higher harmonic signals. However, one should also consider the bandwidth of the analog-to-digital converter (ADC) used in the system, which has a cut-off at about 1 MHz. This makes impossible the registration of the fifth harmonic of the signal if the resonant frequency of the AFM probe is too high. In our experiments, we could detect up to the fifth harmonic of the optical signal from the Si-reference sample by using AFM probes with a resonance frequency of <220 kHz.

The spatial resolution of the method is determined by the tip apex radius of the used AFM probe and can reach a value of <10 nm (Mastel *et al.*, 2018 ▸). The new endstation significantly extends the range of the possible experiments at the beamline, enabling investigations of complex multicomponent materials and hierarchical systems, including energy materials, complex microstructured biomaterials, biological macromolecules such as membrane proteins and protein crystals, hybrid materials, objects of cultural heritage, or minerals with tens of nanametres spatial resolution. FTIR nano-spectroscopy can be used as a single technique or, advantageous in most multi-scale studies, in combination with the other available IR-spectroscopy methods.

In addition to enabling to fuse information from different spatial scales ranging from millimetres over micrometres to nanometres, the acquisition of ‘conventional’ IR spectra from the same or similar samples also helps the interpretation of the observed near-field spectra – IR spectra collected with the s-SNOM can differ significantly from the much more common FTIR spectra obtained using far-field reflectance, transmission and attenuated total reflectance (ATR) methods (Amarie & Keilmann, 2011 ▸; Mastel *et al.*, 2015 ▸; Amenabar *et al.*, 2017 ▸).

As an example, recently we investigated the orientation of cellulose microfibrils in Sorghum using diffraction-limited polarized IR micro-spectroscopy and a preferential orientation of cellulose and other macromolecules at the different scales ranging from the plant tissues to single cell walls (Veber *et al.*, 2023 ▸). Detailed analysis of the anisotropic behavior of the spectra also allowed us to determine the orientation of the cellulose microfibrils indirectly, however, averaged over an area of about 5 µm × 5 µm, corresponding to a diffraction limit at 1160 cm^−1^, the wavenumber of a polarization-sensitive band in the spectrum of cellulose.

**Figure 4:**
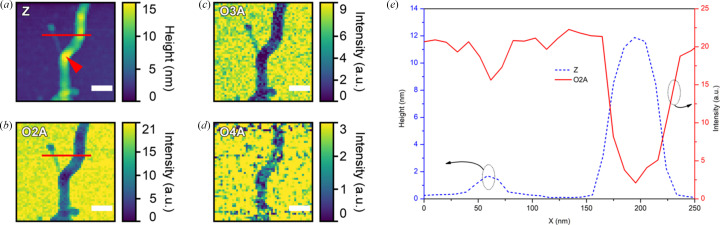
*Z*-profile (*a*) and white-light images at the second (*b*), third (*c*) and fourth (*d*) harmonics of the optical amplitude signal of cotton cellulose deposited on a silicon substrate. The height and the optical signal profile (*e*) are taken along the red line shown in panels (*a*) and (*b*). The arrowhead in panel (*a*) points to the position from where the nano-FTIR spectrum (*cf*. Fig. 5 ▸) was collected. The scale bar in panels (*a*)–(*d*) represents 100 nm. The AFM probe oscillation frequency and the tapping amplitude in the experiment were 259 kHz and 25 nm, respectively.

The use of the IR nano-spectroscopy technique allows investigations significantly below the diffraction limit and to directly visualize individual cellulose microfibrils as nanoscopic material. Multiple signals are recorded by the system simultaneously. Fig. 4 ▸ shows images obtained from cotton cellulose microfibrils deposited on an Si substrate. One can see the topography, the second, the third and the fourth harmonics of the optical amplitude signal in Fig. 4 ▸, panels (*a*), (*b*), (*c*) and (*d*), respectively. According to the topography measurements (*Z*-profile), the width of the smallest microfibril detected in the area is about 30 nm, and its height is less than 2 nm [Figs. 4 ▸(*a*) and 4(*e*), dashed blue trace]. The width is comparable with the specified radius of the AFM probe tip apex of 25 nm that was used in the experiment. This microfibril can be observed in the optical amplitude signal and is clearly visible in the third harmonic of the IR optical amplitude signal in the white-light imaging mode. A larger microfibril of 70 nm width and 12 nm height results in higher contrast in the optical image [Figs. 4 ▸(*a*) and 4 ▸(*e*)] and can be detected up to the fourth harmonic of the optical amplitude signal [Fig. 4 ▸(*d*)].

**Figure 5:**
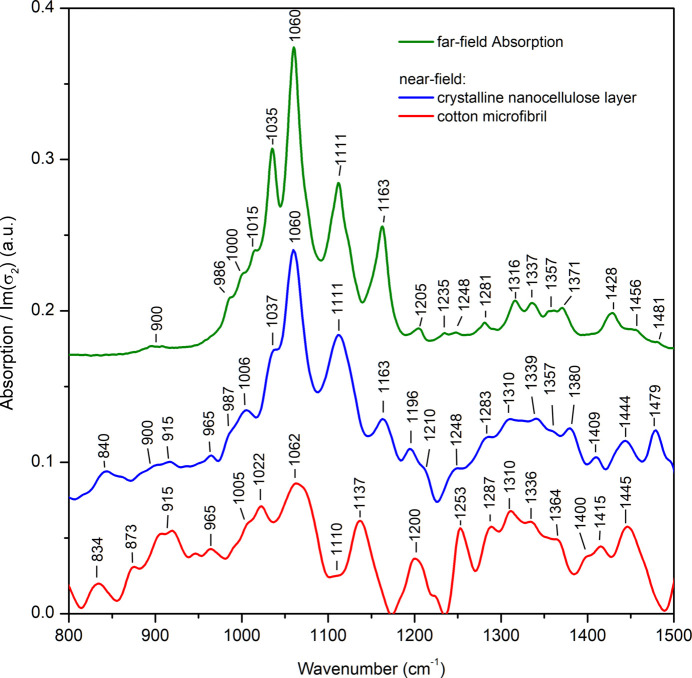
Near-field absorption spectra of a single cotton microfibril (red/bottom trace) and of the thick layer of unoriented crystalline nanocellulose (blue/middle trace) as well as the far-field absorption spectrum of the unoriented crystalline nanocellulose layer (green/top trace). AFM probe oscillation frequency and tapping amplitude were: for the cellulose single microfibril 259 kHz and 25 nm; for the crystalline nanocellulose 248 kHz and 60 nm. The vertical scale for the near-field absorption spectra is the same, whereas the far-field absorption spectrum was scaled arbitrarily to match the intensity of the near-field data. The spectra are shifted vertically for clarity.

The typical integration time per pixel for the imaging mode is about 20–50 ms, allowing quick acquisition of images of rather large sample areas. Despite the observed contrast, these images do not contain exact spectral information and represent intensity changes over the whole broad spectrum reaching the sample, different from the typical chemical imaging that uses single spectral bands or a single-frequency radiation source. To reveal the specific spectral changes at the point of interest, an interferogram is recorded and converted to the spectrum using the FFT algorithm. The corresponding spectrum of the cotton microfibril from an effective volume of about 30 nm × 30 nm × 12 nm is shown in Fig. 5 ▸ (bottom trace). Fig. 5 ▸ also contains near-field (middle trace) and far-field (top trace) absorption spectra acquired from a thick layer of randomly oriented crystalline nanocellulose deposited on a Si surface. The crystalline nanocellulose spectra collected using the s-SNOM technique and standard far-field FTIR spectroscopy are in good agreement. Despite slight frequency shifts of the vibrational bands, a good correlation is also observed between the near-field amplitude and the far-field reflection as well as between the near-field phase and the far-field absorption spectra (see Fig. S1 of the supporting information). This provides evidence that many of the vibrational modes of cellulose originate from weak oscillators, and a comparison of nano-FTIR and far-field FTIR absorptions is applicable (Govyadinov *et al.*, 2013 ▸), which significantly simplifies the interpretation of the standalone near-field spectra in this case.

At the same time, the vibrational bands present in the cellulose microfibril spectrum (Fig. 5 ▸, red/bottom trace) differ significantly from the reference cellulose sample (Fig. 5 ▸, blue/middle trace): the vibrational bands at ∼1110 and ∼1160 cm^−1^ are weak or not present in the collected spectrum of the single cellulose fibril. We found that the near-field spectra recorded for different microfibrils and at different points of the same microfibrils demonstrate quite high variability (Fig. S3); however, the spectra collected in the proximity of the same location are quite reproducible, especially in the range 950–1200 cm^−1^. The single cotton microfibril spectrum is also not unique and similar spectra could be observed at different locations of other cotton microfibrils or microfibril bunches (*cf*. Fig. 5 ▸ and Fig. S2).

Thus, the spectrum of the single cotton microfibril is a sign of changes in the cellulose structure at the location of the measurement, and there are two possible reasons which could explain the observed spectral features. The band at 1111 cm^−1^ is ascribed to a ring-stretching vibration and the intensity of this band is known to depend on the crystallinity of the cellulose. The absence of a band at 1111 cm^−1^ has been interpreted as evidence of amorphous cellulose (Nelson & O’Connor, 1964 ▸), and it has been confirmed using both far-field and near-field FTIR spectroscopy techniques (Nelson & O’Connor, 1964 ▸; Kotov *et al.*, 2023 ▸). However, the amorphous cellulose should also demonstrate significant changes in other vibrational bands; in particular, the bands should become broader, and the intensity of the spectral bands at 1315 and 1336 cm^−1^ should decrease significantly when compared with highly crystalline cellulose. Also, this hypothesis does not explain the absence of the band at 1163 cm^−1^, which was previously observed in both amorphous and crystalline cellulose using the nano-FTIR technique (Kotov *et al.*, 2023 ▸).

It is known that the tip-enhanced s-SNOM method is very sensitive to the anisotropy of the sample, due to its higher sensitivity to out-of-plane vibrational modes (Muller *et al.*, 2016 ▸). In the experiment here, the single microfibril is oriented normally to the AFM probe, *i.e.* the relative contribution of the modes normal to and in the direction of the cellulose chain should increase and decrease, respectively, when compared with the unoriented reference crystalline nanocellulose sample. This could indeed explain the absence of the characteristic peak at 1163 cm^−1^ in the single fibril spectrum since this band is ascribed to the asymmetric C–O–C stretching vibration in the glycosidic linkage and directed in plane in our s-SNOM experiment. Likewise, and in accord with this interpretation as well, the bands at 1200, 1253, 1310 and 1336 cm^−1^, ascribed to the C–O–C symmetric stretching of the glycosidic linkage, the CH_2_ wagging mode, an antisymmetric C–H deformation and the OH in-plane deformation, respectively (Tsuboi, 1957 ▸; Liang & Marchessault, 1959 ▸), correspond to the out-of-plane modes in our experiment, resulting in the more pronounced bands at these frequencies in the single fibril spectrum. Similar changes in the relative intensities of the cellulose vibrational bands in the 1200–1500 cm^−1^ energy region are observed in the far-field polarized absorption spectra of oriented cellulose collected with the IR-light polarized along and perpendicular to the cellulose chain axis (see Fig. S3): as for the single fibril near-field spectrum the bands at ∼1200 and ∼1250 cm^−1^ in the far-field spectrum are only visible at 90° orientation of the crystal as well as the bands at 1315 cm^−1^ and 1335 cm^−1^ being much more pronounced at this orientation (see Fig. S3).

Thus, the single cotton microfibril at the location of measurement is probably less crystalline and differently oriented when compared with the reference cellulose sample. The overall variability between the observed spectra agrees well with the results obtained by Kotov *et al.* (2023 ▸) and can be explained by the heterogeneous organization of the microfibrils at the ∼30 nm spatial scale, in particular a local change of the cellulose orientation and crystallinity even within a single microfibril. Further extensive work is currently being conducted to verify this using the IR s-SNOM method. In the present work, we would like only to demonstrate that the s-SNOM method is very sensitive and can be used for investigation of the nanoscale objects.

## 4. Optimization of the beamline performance

**Figure 6:**
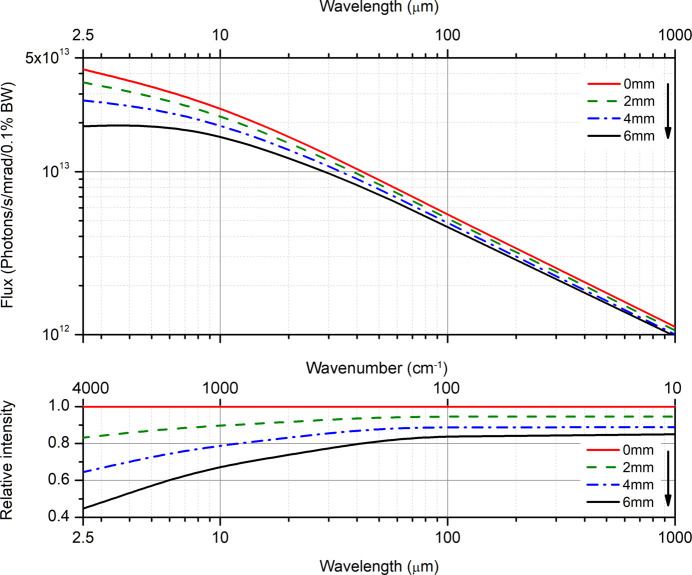
Absolute flux (top panel) and relative intensity (bottom panel) after the M1 mirror versus wavelength/wavenumber calculated for different slot sizes.

The spectral range of the beam delivered to the endstations is a function of the emission of the light source, the acceptance angle of the M1 mirror, the slot size of the mirror, and the transmission function of the beamline optics. The maximum flux after the M1 mirror, assuming no slot in the mirror, increases monotonously with the frequency of the emitted light (Fig. 6 ▸). However, the slot allows high-energy X-rays and UV radiation to pass through the mirror towards an absorber behind the mirror. Thus the slot is important to decrease the thermal load on the M1 mirror. Since the IR beamline was commissioned shortly after the start of the synchrotron operation, the slot was set to 6 mm, to protect the mirror from possible instabilities of the electron beam positions and the subsequent hit by the high-energy radiation in such a case. However, the current configuration significantly decreases the flux at shorter wavelengths, so that, for example, only about 50% of the radiation from the maximal power is transmitted at λ = 2.5 µm (4000 cm^−1^). To date we have gained thorough information about the beam stability and the slot size can be decreased down to 2 mm, which will significantly increase the flux at wavelengths < 10 µm (wavenumbers > 1000 cm^−1^). This modification is currently at the preparatory stage.

The spectral response at the different endstations can also be modified by utilizing an appropriate IR detector. The same detectors can be used at FTIR spectroscopy and microscopy endstations; in particular, Si-bolometers are used to measure in the far-IR spectral region. Unfortunately, the long response time of commonly used Si- as well as transition-edge superconducting bolometers does not allow their utilization for the s-SNOM technique, where oscillations with frequencies up to 1 MHz need to be detected. Currently, we are working on the optimization and coupling of a liquid-helium-cooled Ge:Cu fast photoconductive detector to the nano-spectroscopy endstation, which will ensure sensitivity in the 300–600 cm^−1^ spectral range (Khatib *et al.*, 2018 ▸). Moreover, a higher band-gap MCT detector will be added to the system to increase the signal in the range 1500–4000 cm^−1^.

**Figure 7:**
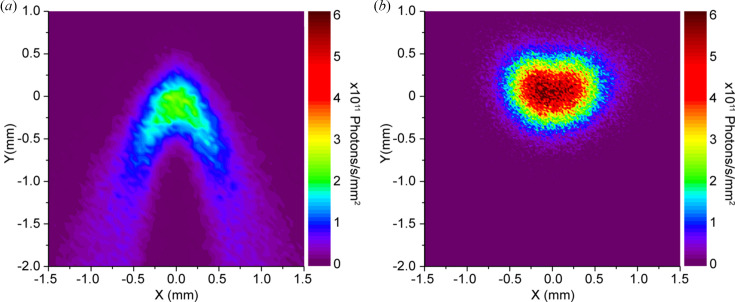
Calculated beam profile at λ = 10 µm (1000 cm^−1^) for the current beamline optical scheme after the M5 mirror (*a*) and after using the potential low-aberration optical scheme (*b*), when the beam is scaled to the same dimensions as in the case of (*a*).

During the upgrade, we also considered the possibility of modifying the front-end of the beamline to improve the beam quality. The front-end of the beamline uses a traditional source-refocusing concept. This optical scheme cannot completely compensate for the aberrations caused by the depth and the circular source shape resulting from the electrons trajectory (Moreno *et al.*, 2013 ▸) and results in a non-circular shape of the beam in focus [see Fig. 7 ▸(*a*)]. This can be corrected using a so-called magic mirror (López-Delgado & Szwarc, 1976 ▸) or a more recent optical outline proposed by Moreno *et al.* (2013 ▸).

Our ray-tracing calculations show that an optical scheme made according to the Moreno concept applied to the IRIS beamline would result in a much more circular beam profile in focus, and the central spot maximal intensity increases by about 2.5 times [see Fig. 7 ▸(*b*)]. The parameters used for the ray tracing are described in Section S1 of the supporting information; the 3D model and a side view of the proposed low-aberration optical layout are shown in Fig. S4.

Despite the significant benefit in the beam quality, the realization of the low-aberration port cannot be done by a simple exchange of the mirrors but would require a major modification of the beamline front-end. Thus the adapted Moreno concept could be used as a basis for the design of the new IR beamline at the planned fourth-generation BESSY III synchrotron (Goslawski *et al.*, 2022 ▸), but will not be implemented at the current IR beamline.

The acquisition time of a single interferogram using the nano-spectroscopy endstation depends on many factors; in particular, the sample origin, the AFM-probe tip radius, the probed volume/thickness of the sample, the desired resolution, and the spectral range, to mention just a few. For biological samples, like the single cellulose microfibrils discussed above, acquisition of a good quality spectrum could require up to 0.5–1 h of integration time. This requires extreme mechanical stability of the AFM microscope positioning system. Our tests show that the drift of the AFM varies in the range 1–5 nm min^−1^ [Fig. S5(*a*)], which complicates the accumulation of the spectral data from a single spot for a very long time. Temperature stability of the system is at least one reason for this behavior. Typical daily temperature variations of our system do not exceed 1 K [Fig. S5(*b*)]. To overcome the shift issue one can perform a series of alternating rather quick interferometer scans with the quick imaging of the sample area of interest. The images can be used to track the shift of the sample and correct the position of the AFM probe for the next interferogram scan. Determination of the shift can be done by using any of the recorded AFM-signals, that results in a high contrast image, by calculating the cross-correlation between two subsequent images. We found that such an algorithm allows us to determine the shift with a precision better than one pixel between the two subsequently recorded images and that mechanical amplitude and phase signals are usually well suited for this purpose. Currently, we are working on the integration of the AFM-drift correction software into the control software of the near-field microscope.

Recently it has been shown that the performance of the FTIR microscope can be significantly improved by optimization of the beam profile at the endstation using a deformable mirror (Kalkhoran *et al.*, 2022 ▸). We believe that this concept can also be applied to improve the coupling of the synchrotron beam to the AFM probe of the s-SNOM. For comprehensive vibrational spectroscopic imaging of identical samples in an identical microscope, we are currently developing a combined Raman/IR microscope, which will open further possibilities in multimodal microscopy at the beamline.

## 5. Summary

During the upgrade, the IR beamline IRIS at the BESSY II storage ring was extended and now consists of four endstations: IR (1) macro-, (2) single-shot time-resolved, (3) micro- and (4) nano-spectroscopy. In this summary, we show the current outline of the beamline and discuss the available experimental methods. Overall, the imaging and spectroscopy capabilities have been improved by enabling a higher imaging rate using an FPA detector for diffraction-limited IR microscopy and by extending the spectral range of the diffraction-limited experiments to the far-IR spectral region. The nano-spectroscopy endstation is based on a scattering-type scanning optical microscope, which enables IR imaging and spectroscopy with a spatial resolution better than 30 nm. The performance of this new endstation is exemplified here by the investigation of single cellulose microfibrils. All the end­stations are available for national and international user groups. We are constantly developing the beamline to ensure the availability of state-of-the-art IR-spectroscopy techniques to the users.

## 6. Sample preparation and data analysis

The sample for an investigation of individual cellulose microfibrils was prepared using a cellulose nanofibrils slurry (3 wt%) from cotton (Cellulose Lab Inc., Canada), which was diluted with water to a nominal concentration of 5 × 10^−4^ wt% and drop-cast onto a clean Si-substrate and dried in air.

Purified crystalline Iβ nanocellulose from trees (NAVITAS, Slovenia) was used to prepare reference cellulose samples. A thin layer of this material was obtained by blade casting of the cellulose slurry on a Si substrate resulting in a typical thickness of the film of about 3 µm. This sample was used to record far-field FTIR transmission and near-field SNOM reference spectra of cellulose. Repetitive blade casting was performed to obtain an optically thick layer of the cellulose for the far-field FTIR reflection measurement.

Pt-coated Si AFM tips with an apex radius of ∼25 nm (Arrow NCPt, Nanoworld) were used for the near-field IR imaging and spectroscopy. The integration time for white-light imaging mode was 20 ms per pixel. The typical time used to collect a nano-FTIR spectrum from a cellulose microfibril was 17 min (an average of ten scans, 1.7 min each acquired in a row). In the case of the single microfibril measurement, to minimize the drift effect, we collected separately 16 spectra of about 3.5 min each and performed imaging between the interferometric scans to ensure the correct positioning of the AFM tip.

The *Gwyddion* (Nečas & Klapetek, 2012 ▸) open-source software was used for the analysis and visualization of the images obtained by the s-SNOM. The interferograms recorded by the s-SNOM were processed using an in-house-developed script in *SciLab* open-source software using a three-term Blackmann–Harris apodization function window and zero-filling factor of four. Ray tracing of the beamline optical path was performed using *SpotX* software (Moreno & Idir, 2001 ▸). Plotting of the data was carried out using *OriginPro* software (Origin­Lab, Northampton, MA, USA).

## 7. Related literature

The following reference, not cited in the main body of the paper, has been cited in the supporting information: Moreno (2017 ▸).

## Supplementary Material

Section S1 including Figs. S1 to S5. DOI: 10.1107/S1600577524002753/ok5108sup1.pdf

## Figures and Tables

**Table 1:** List of endstations available for user operation at the IRIS beamline and their main characteristics

Endstation	Equipment used	Spectral range (cm^−1^)	Spatial resolution at λ = 10 µm (ω = 1000 cm^−1^)	Available modalities	Special sample environment
(1) IR macro-spectroscopy	Bruker Vertex 70 V	2–10000†	≥ 1 mm	Transmission; specular/diffuse reflection; ATR; polarimetry, including photoelastic modulator (PEM); rapid-scan (15 ms) and step-scan (5 ns); far-IR microscopy	Vacuum; *T* = 4–800 K; controlled gas/humidity atmosphere
(2) Single-shot spectroscopy	Dispersive Fery prism spectrometer	1000–1800	∼1 mm	5 µs time resolution; transmission; photoexcitation at 532 nm.	
(3) IR micro-spectroscopy	Bruker Vertex 80 + Hyperion 3000	80–5000	∼6 µm	Transmission; reflection; ATR; polarimetry, including PEM; FPA (64 × 64 pixels) hyperspectral imaging; rapid-scan (10 ms) and step-scan (5 ns)	*T* = 77–500 K; diamond compression cell
(4) IR nano-spectroscopy	neaScope scattering-type near-field optical microscope	600–2000‡	≥ 25 nm	Reflection; white-light imaging; FTIR spectroscopy; PsHet imaging§	Under development

† The spectral range 2–20 cm^−1^ is available in the low-α operational mode of the storage ring only.

‡ Detector limited and is planned to be extended to 330–4000 cm^−1^ in early 2024.

§ PsHet is a laser-source-based modality and is available in the spectral range 1675–1865 cm^−1^.
