# Supplementary material for: Infrared spectroscopy across scales in length and time at BESSY II
Source: J Synchrotron Radiat. 2024 Apr 23;31(Pt 3):613–21. doi: 10.1107/S1600577524002753 (PMC11075711; doi:10.1107/S1600577524002753)
Supplement: Supplementary file 1 [file s-31-00613-sup1.pdf]

JOURNAL OF SYNCHROTRON RADIATION

Volume 31 (2024)

Supporting information for article:

Infrared spectroscopy across scales in length and time at BESSY II

Alexander, Alexander Veber, Ljiljana Puskar, Janina Kneipp and Ulrich Schade

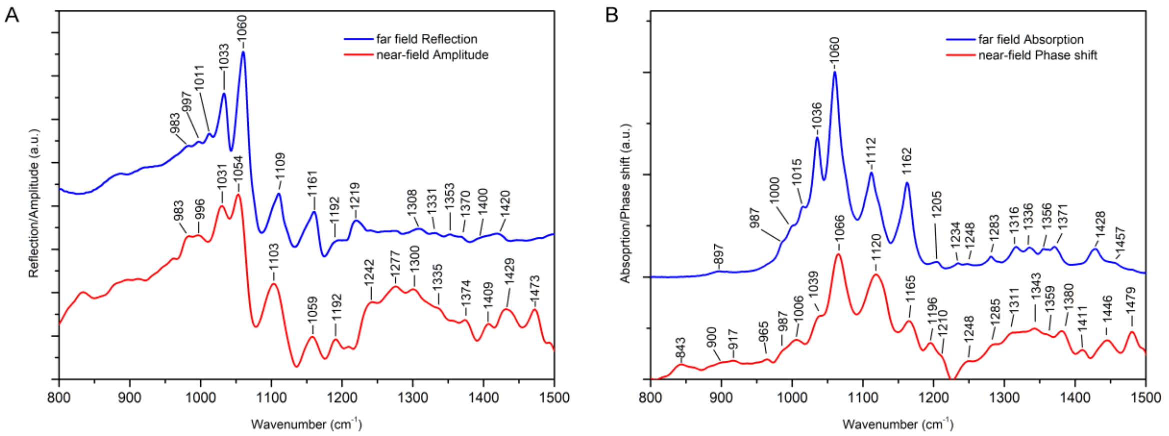

Figure S1 Comparison of the crystalline nano cellulose far-field absorption and reflection spectra with near-field amplitude and phase signal spectra. The second harmonic of the near-field optical signal was used for the amplitude and phase spectra.

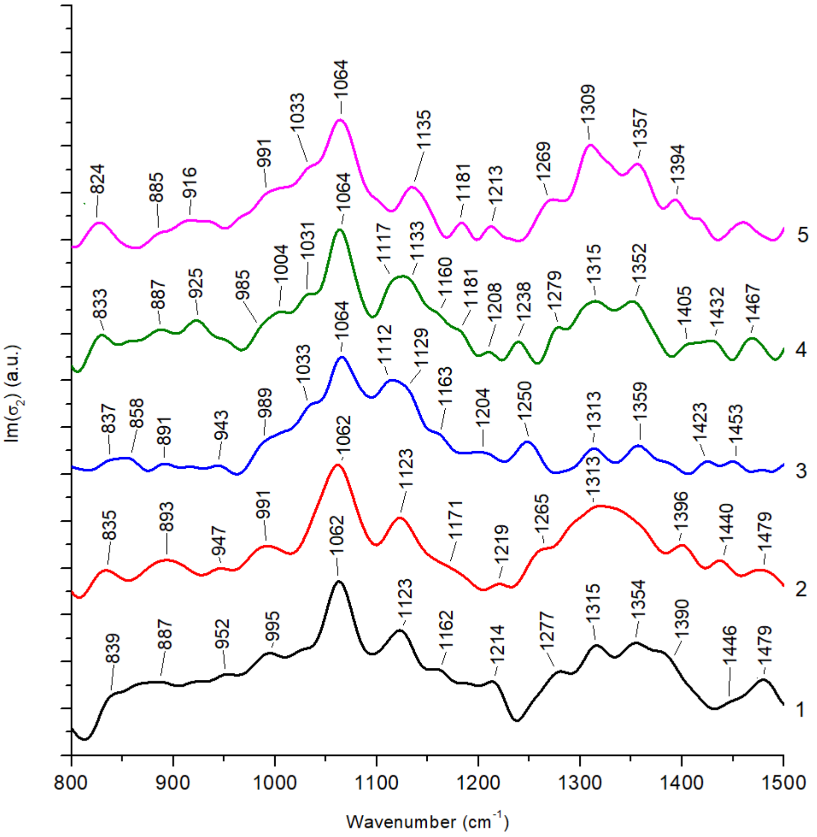

Figure S2 Near-field absorption spectra collected from different cotton microfibrils. Spectra 1 and 2 were collected from the same point of interest – the distance between the two measurement points does not exceed 50 nm. The second harmonic of the near-field optical was used for the spectra.

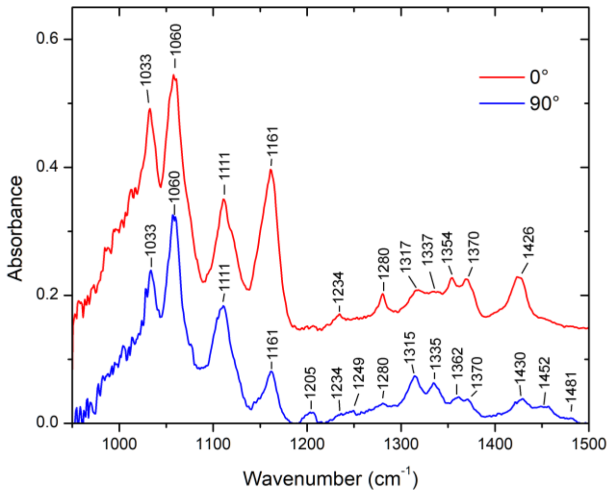

Figure S3 Polarized far-field absorption spectra recorded from a highly oriented cellulose single microcrystal. The infrared light was oriented along and perpendicular to the cellulose chain axis at 0° and 90° spectra, respectively. The zero-degree spectrum is shifted vertically for clarity. The spectra are reproduced from our recent work (Veber *et al.*, 2023).

S1. Moreno concept considerations

The Moreno concept allows one to solve the depth and the circular source aberration by use of a conical mirror and a long focal length cylindrical mirror. The exact focal length and shape of the mirrors are unambiguously determined by the bending magnet source parameters, position, and orientation of the mirrors. To compensate for the aberrations all these parameters have to satisfy a set of equations (Moreno, 2017).

For the ray tracing calculations shown in this work, the cylindrical mirror M2 was replaced with a conical mirror with a radius of 1910 mm at the center and a radius gradient of 1.9 mm/mm, the position and the orientation of the mirror did not change. The Moreno style scheme cannot be implemented by simple replacement of the current M3 with a new long focal length cylindrical mirror, since the current position of M3 is too close to the dipole magnet. To implement the aberration-free outline, the current cylindrical M3 mirror can be replaced with a flat mirror, and an additional cylindrical mirror (M*), determined in terms of the Moreno concept, is installed at a distance of about 3 m from the source, and M* could redirect the beam towards the storage ring wall (instead of the roof), where a new port would be required. At this position and the AOI of 45° M* needs to have a focal length of about 15 meters. To decrease the focal length of M* mirror the AOI on both new flat M3 and M* can be decreased to 35°, which results in M* radius of 6670 mm and the intermediate focus at about 5.2 m from M*. The 3D model and side view of the proposed low-aberration optical layout are shown in supporting information in Figure S4.

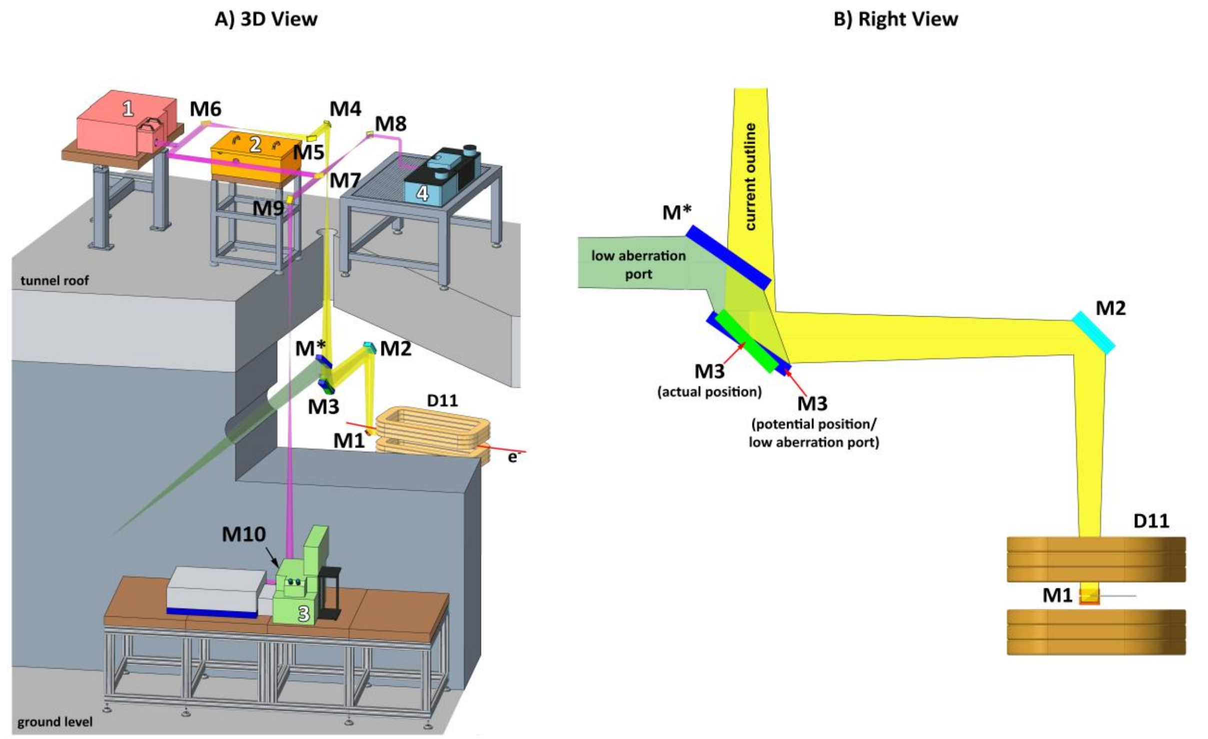

Figure S4 Outline of potential implementation of the Moreno style low aberration optical port at the IRIS beamline: A) 3D model and B) Side view of the front-end (M1-M3 mirrors) of the IRIS beamline optical scheme. The yellow beam, redirected upwards with M3 (actual position) corresponds to the current optical scheme, and the light green beam, redirected (sidewards) with M*, corresponds to the potential low aberration extraction port.

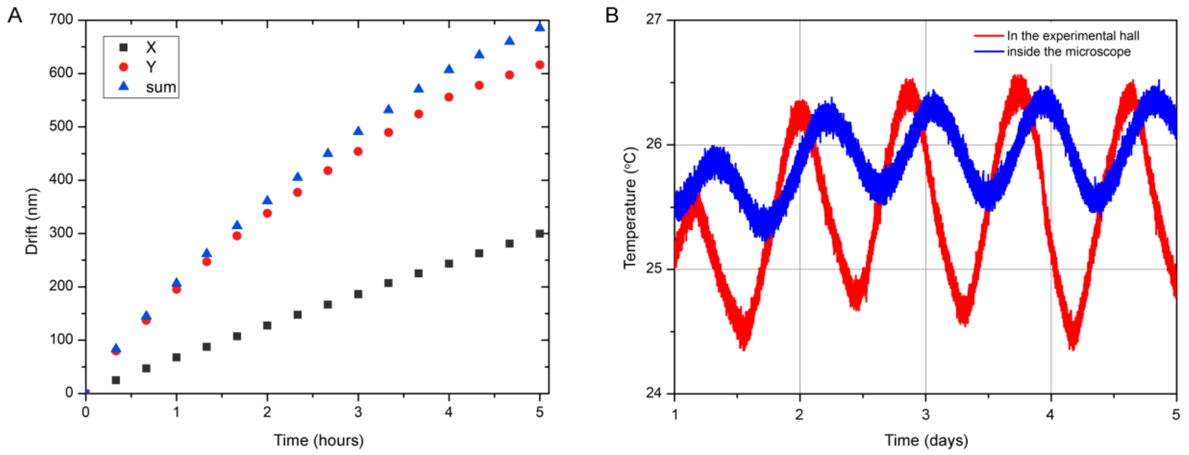

Figure S5 A) Drift of the AFM probe tip position is time in horizontal (X), vertical (Y) direction and the total value (sum). B) Variation of temperature in the experimental hall of the BESSY II storage ring (red curve) and inside the enclosure of the nano-spectroscopy end-station (blue curve). The day-night temperature variation in Berlin was about 10 °C in the days of the temperature logging.
